# TIGIT-based immunotherapeutics in lung cancer

**DOI:** 10.1093/immadv/ltad009

**Published:** 2023-05-26

**Authors:** Akshay J Patel, Gary W Middleton

**Affiliations:** Institute of Immunology and Immunotherapy, University of Birmingham, Birmingham, UK; Department of Thoracic Surgery, University Hospitals Birmingham, Birmingham, UK; Institute of Immunology and Immunotherapy, University of Birmingham, Birmingham, UK; Department of Medical Oncology, University Hospitals Birmingham, Birmingham, UK

**Keywords:** TIGIT, lung cancer, non-small cell lung cancer, small cell lung cancer, immunotherapy

## Abstract

In this review, we explore the biology of the TIGIT checkpoint and its potential as a therapeutic target in lung cancer. We briefly review a highly selected set of clinical trials that have reported or are currently recruiting in non-small cell and small cell lung cancer, a disease transformed by the advent of PD-1/PD-L1 checkpoint blockade immunotherapy. We explore the murine data underlying TIGIT blockade and further explore the reliance of effective anti-TIGIT therapy on DNAM-1(CD226)-positive activated effector CD8+ T cells. The synergism with anti-PD-1 therapy is also explored. Future directions in the realm of overcoming resistance to checkpoint blockade and extending the repertoire of other checkpoints are also briefly explored.

## Graphical Abstract

**Graphical Abstract:**
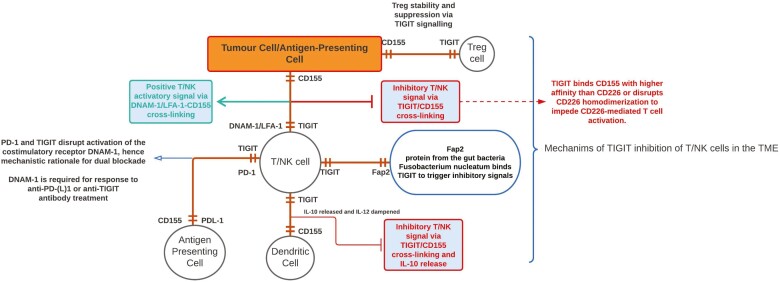

## Introduction

The advent of checkpoint blockade immunotherapy has revolutionised the management of solid cancers since its inception. Targeting the PD1/PD-L1 checkpoint has transformed the landscape of lung cancer and melanoma in both the early and advanced disease setting [1, 2]. Tumour evolution strategies include ways in which to evade the host immune response, which is a causal link with immunotherapy resistance [3, 4]. Research into further immunotherapeutic candidates is currently thus at the forefront of cancer research; one such candidate molecule is ‘T cell immunoglobulin and ITIM domain’ (TIGIT). This is an inhibitory receptor expressed on lymphocytes that interacts with its complementary target, CD155 (Polio Virus Receptors, PVR or NECL-5) on the surface of antigen-presenting cells or tumour cells to suppress T and natural killer (NK) cell anti-tumour responses [5]. CD155 acts as a ligand for DNAM-1 (CD226) and CD96, in addition to TIGIT, which possesses the highest affinity [6]. Cross-linking of DNAM-1 and CD155 results in a cytotoxic lymphocyte stimulation, murine data has shown that DNAM-1 knockout mice demonstrate poor CD8+ T and NK cellular responses with poor tumour cell elimination and accelerated tumour growth [7, 8]. TIGIT knockout results in the loss of anti-tumour T and NK cell suppression, and this has been shown in murine models, without the added burden of high-grade autoimmune sequelae [9].

## TIGIT structure, function, and expression

TIGIT is a PVR-like protein possessing an intracellular domain, an extracellular variable immunoglobulin (Ig) domain, a type I transmembrane domain, and a cytoplasmic tail with two inhibitory motifs that are conserved in both mouse and humans: an immunoreceptor tyrosine-based inhibitory motif (ITIM) and an Ig tail-tyrosine (ITT)-like motif [5, 10]. These motifs have been shown to mediate recruitment of the phosphatase SHIP-1 following ITT-like motif phosphorylation at Tyr225 with resultant binding to cytosolic adaptors Grb2 and β-arrestin 2. This results in dampened phosphoinositide 3 kinase and mitogen-activated protein kinase signalling as well as inhibition of TRAF6 and NF-κB activation, thus providing a mechanism by which TIGIT can act cell intrinsically to dampen activating signals [11].

The Ig variable domain shares sequence homology with DNAM-1, CD96, CD155, and other PVR-like proteins [10]. Structural analysis of TIGIT bound to CD155 reveals that two TIGIT/CD155 dimers assemble into a hetero tetramer with a core TIGIT/TIGIT cis homodimer, with each TIGIT molecule binding to one CD155 molecule. This cis–trans receptor clustering mediates cell adhesion and signalling [12, 13].

Expression is seen on T cells (CD4+, CD8+, and T_regulatory_ (T_regs_) cells) and NK cells, and this can be upregulated upon cellular activation [10]. In contrast to DNAM-1, TIGIT is weakly expressed by naive T cells; in cancer, it is co-expressed with PD-1 on tumour antigen-specific CD8+ T cells and CD8+ tumour-infiltrating lymphocytes (TILs) in humans. On exhausted tumour-specific CD8+ T cell subsets, TIGIT co-expresses with other inhibitory receptors, LAG3 and TIM-3 [14].

Foxp3+ T_regs_ are well known to be suppressive components of the adaptive immune response, and recent data in melanoma has demonstrated that T_regs_ exhibit increased expression of TIGIT, and decreased expression of its competing costimulatory receptor DNAM-1 as compared with CD4+ T cells resulting in an increased TIGIT/DNAM-1 ratio. TIGIT+ T_regs_ are highly suppressive and enriched in tumours and correlated with poor clinical outcome upon checkpoint blockade [15]. The high expression levels of coinhibitory receptors such as TIGIT on T_regs_ is associated with their potent immunosuppressive function, as such understanding these co-inhibitory receptors on effector T cells and T_regs_ is vitally important to developing new treatment strategies for cancer and indeed other chronic diseases. TIGIT+ T_regs_ represent a highly active and suppressive phenotype with TIGIT signalling driving this phenotype with suppression of anti-tumour immunity being mediated via these T_regs_ and not CD8+ T cells [16].

**Figure 1.**
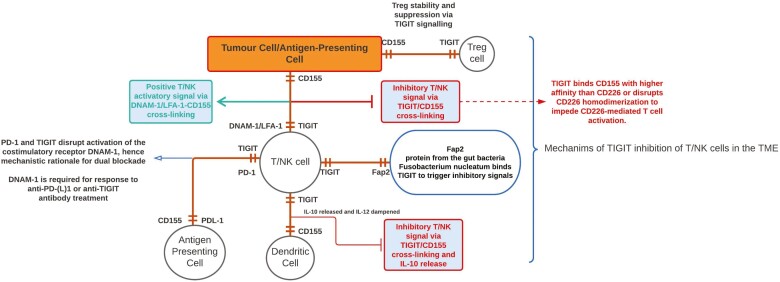
Interplay in the TIGIT/CD155/DNAM-1 axis.

TIGIT acts by binding with CD155 (cellular extrinsic mechanism), by interfering with DNAM-1 co-stimulation or by delivering direct inhibitory signals to effector cells (cellular intrinsic mechanism) [5]. CD155 cross-linking with TIGIT results in a shift of the cytokine milieu whereby dendritic cells change from a pro-inflammatory IL-12 centric to a suppressive IL-10 centric phenotype [10]. This polarisation switch has also been demonstrated in CD155 expressing macrophages that change from a type I proinflammatory phenotype to a type II suppressive phenotype [17]. TIGIT+ T_regs_ are more efficacious in their ability to suppress Th1 and Th17 responses than TIGIT- T_regs_ [18]. Figure 1 illustrates the interplay and mechanisms of suppression in the TIGIT/CD155/DNAM-1 axis.

## Pre-clinical murine data in solid tumours

TIGIT-deficient mice show significantly delayed tumour growth in distinct murine models [19]; however, the number of pulmonary metastatic deposits was comparable following B16 melanoma cell inoculation in TIGIT-deficient and wild-type mice [16, 20]. Conversely, Zhang’s group reported that TIGIT deficiency protected mice against secondary pulmonary metastasis [19]. TIGIT expression on CD8+ TILs is often correlated with other inhibitory receptors such as PD-1, lymphocyte-activation gene 3 (LAG-3), T-cell immunoglobulin, and mucin-domain containing-3 (TIM-3), and with decreased expression of DNAM-1 [5, 8, 16, 20]. B16F10 or RM-1 cell line inoculated TIGIT knockout mice further treated with anti-TIM-3 monoclonal antibodies have demonstrated further regression of lung metastasis compared to wild-type receiving the same treatment suggesting synergism between the checkpoints [16]. This has been further illustrated in the MC38 murine model, where co-blockade of TIGIT and PD-1 enhanced the effector T cell responses more than each therapy individually and led to a 100% cure rate in these mice [21]. This has been further elicited in CT26 inoculated murine models with co-PD-L1 and TIGIT blockade, which resulted in enhanced CD8+ T cell effector responses and a 75% decrease in mean tumour volume after 16 days of treatment, significantly higher clearance compared to individual receptor targeting alone (*P* < 0.0001) [22].

## TIGIT expression in human lung cancer

Meta-analytical data [23] from 1426 cancer patients, demonstrated that high TIGIT expression is a poor prognosticator for overall survival in solid cancers (HR 1.66, 95% CI [1.26–2.20], *P* < 0.001). Subgroup analyses in lung cancer patients showed the same prognostic relationship (HR 1.29, 95% CI [0.96–1.72], *P* = 0.094), albeit not significant. Retrospective data from resected lung squamous cell carcinoma specimens has shown through immunohistochemistry that 85.8% of samples expressed CD155 (PVR), compared to 26.8% expressing PD-L1 [24]. High TIGIT density and high CD155/TIGIT expression correlated with advanced Tumour/Nodal/Metastasis (TNM) stage (*P* = 0.02 and *P* = 0.04, respectively) and significantly worse overall survival (*P* = 0.027 and *P* = 0.014, respectively). Similar findings have been shown in independent cohorts [25]. CD155/PD-L1 co-expression is a significantly independent negative prognostic factor in squamous cell carcinoma (HR 1.76, 95% CI [1.152-2.676], *P* = 0.009) [24]. CD155 and TIGIT expression has been correlated with significantly shorter overall and progression-free survival (PFS) in lung adenocarcinoma [26], and CD155 additionally has demonstrated this in small-cell lung cancer (SCLC) [27]. TIGIT expression on tumour-infiltrating lymphocytes (TILs) in non-small cell lung cancer (NSCLC) is upregulated when compared with healthy controls, but moreover, the expression dynamics of TIGIT exceed that of PD-1 in that TIGIT mRNA increased more rapidly than PD-1 mRNA, and TIGIT+ CD8+ T cells upregulated PD-1 more rapidly than TIGIT- CD8+ T cells in NSCLC patients [28]. T_regs_ are well-recognised suppressors of anti-tumour effector T cell responses; CD4+ CD25+ FoxP3+ TIGIT+ T_regs_ were significantly elevated in the bronchoalveolar lavage fluid and peripheral blood of NSCLC patients compared to healthy control subjects [29]. The data presented intimates the role of TIGIT as a contributor to lung cancer progression and a candidate biomarker for immunotherapeutic targeting.

## Targeting TIGIT in lung cancer

The paradigm of anti-TIGIT and anti-PD-1/PD-L1 synergism exerting better tumour control and overall survival was taken forward in a large phase II trial; CITYSCAPE [30] which reported in early 2022. Patients with locally advanced or metastatic NSCLC expressing PD-L1 in at least 1% of tumour cells and without eGFR or ALK alterations were enrolled and randomised to receive Tiragolumab (anti-TIGIT monoclonal antibody) plus Atezolizumab (anti-PD-L1) (*n* = 67) or Atezolizumab alone (*n* = 68). The co-primary endpoints were investigator-assessed objective response rate (ORR) and PFS as per Response Evaluation Criteria in Solid Tumours version 1.1 criteria (RECIST) [31] in the intention-to-treat population. At 5.9 months of median follow-up, 21 patients (31.3%) in the combination arm versus 11 patients (16.2%) in the anti-PD-L1 arm had an objective response (*P* = 0.031). Median PFS was 5·4 months (95% CI 4.2–not estimable) in the combination arm versus 3·6 months (2.7–4.4) in the anti-PD-L1 arm (stratified hazard ratio 0·57 [95% CI 0.37–0.90], *P* = 0.015). Patients with a high tumour proportion score (TPS) of PD-L1 expression (>50%) (*n* = 29), the differences between the treatment groups were more pronounced. ORR was 69% and 24.1% in the combination and anti-PD-L1 arm alone, respectively. In contrast, patients in the combination arm with lower PD-L1 expression (1–49%) had an ORR of 16% and a median PFS of 4 months, compared with 18% and 3.6 months in the anti-PD-L1 arm, suggesting the benefit of synergism is driven by PD-L1 expression. Grade 3–4 treatment-related adverse events (TRAE) were seen at a similar frequency in both arms (22.4% vs. 25% for combination versus anti-PD-L1 alone, respectively) as well as grade 3–4 immune-related adverse events (19.4% vs. 16%) [30]. In the phase 3 Impower110 trial [32], atezolizumab showed a significant and clinically meaningful survival benefit over chemotherapy in previously untreated patients with advanced NSCLC with high PD-L1 expression. The preliminary efficacy and safety of Tiragolumab plus atezolizumab as a first-line treatment in patients with PD-L1 high (TPS ≥50%) NSCLC observed in this phase 2 study is being confirmed in an ongoing phase 3 study (SKYSCRAPER-01; NCT04294810) which is expected to enroll 560 patients. The results of this trial are encouraging and further biologically interrogation and application are warranted to explore the benefit in other groups. Pre-clinical data has suggested that DNAM-1 expression maximises the impact of TIGIT blockade, and this may correlate with class I expression [33]. Banta *et al*. [33] have demonstrated a requirement of DNAM-1 expression in order to elicit maximum responses to anti-PD-1 or anti-TIGIT treatment in murine cancer models. There is differential expression of DNAM-1 and CD28 on CD8+ TILs which are regulated by the PD-1/PD-L1 and TIGIT/CD155 checkpoints, respectively. Mechanistically, PD-1 inhibited phosphorylation of both DNAM-1 and CD28 via its ITIM-containing intracellular domain (ICD); with TIGIT restricting DNAM-1 co-stimulation by blocking interaction with their common ligand PVR (CD155). Thus, full restoration of DNAM-1 signalling, and optimal anti-tumour CD8+ T cell responses, requires blockade of TIGIT and PD-1, providing a biological rationale for combinatorial targeting in the clinic [33]. DNAM-1^LO^ CD8+ T cells accumulate at the tumour site and upon interaction with cognate antigen exhibit an exhausted phenotype, expressing high levels of PD-1, LAG-3, TIM-3, and TIGIT [34]. However, DNAM-1^HI^ CD8+ T cells exhibit a greater capacity for self-renewal and responsiveness; it is these high expressors that are most sensitive to anti-TIGIT blockade with subsequent DNAM-1 phosphorylation at tyrosine 322 [34]. Direct antibody-mediated activation of DNAM-1 on these cytotoxic T cells augments the effect of TIGIT blockade on CD8+ T cell responses in pancreatic ductal adenocarcinoma [34]. Murine data has shown CD8+ T cell loss of DNAM-1 at the tumour microenvironment (TME) occurs in a Eomes-dependent manner but importantly limits the efficacy of checkpoint blockade [35]. Dysfunctional DNAM-1-negative CD8+ T cells accumulated in these tumours, and despite expression of co-inhibitory receptors, these cells failed to respond to anti-PD-1 treatment in the absence of DNAM-1 [35]. Enhanced CD8+ T cell effector function as a result of co-PD-1 and TIGIT blockade was abrogated by subsequent DNAM-1 blockade *in vivo* [22]. CD155 ligation with DNAM-1 results in its phosphorylation at Tyrosine 319 by Src kinases enabling its ubiquitination and clearance from the cell surface, and indeed specific mutations such as Y319F prevent phosphorylation and degradation of DNAM1, and thus enable synergism with anti-PD-1 and enhanced anti-tumour immunity in murine models (MC38 and B16) [36]. This effectively drives resistance to checkpoint blockade, and in melanoma, the efficacy of treatment is reliant on DNAM-1+ CD8+ T cells. Mutations of Tyrosine 319 maintain DNAM-1 expression with improved anti-tumour immunity [36]. PD-1 and TIGIT double-positive T cells are predictive of enhanced anti-PD-1 activity as has been shown in melanoma and merkel cell carcinoma [37], but without the presence of DNAM-1 (marking the activation state of CD8+ T cells), there is no durable response to anti-PD-1 or indeed anti-TIGIT blockade (Fig. 1).

First in-human phase I data comparing the anti-TIGIT monoclonal antibody, Vibostolimab alone or in combination with anti-PD-1 agent Pembrolizumab has been reported in solid cancers, including NSCLC. The combination therapy was well tolerated and deemed safe in NSCLC patients with early data showing objective tumour response [38]. Thirty-nine anti-PD-1/PD-L1 therapy naive patients with NSCLC received combination therapy and demonstrated an ORR of 26% with a median PFS of 5 months (95%CI 2–8) [38]. In patients with anti-PD1 refractory NSCLC, activity was negligible both with single agent or combination (ORR 6% and 3% each) [38]. Toxicities were manageable both as a single agent or in combination with Pembrolizumab [38, 39]. No dose-limiting toxicities were seen. Three phase 3 clinical trials investigating combinations of anti-TIGIT and anti-PD-L1 or anti-PD-1 in patients with NSCLC are currently underway (NCT04738487, NCT04746924, and NCT04736173). Other phase I/II studies are underway in lung cancer exploring combinations of humanised IgG1 anti-TIGIT (Domvanalimab/AB154) in combination with anti-PD1 agent Zimberelimab (EDGE-Lung, NCT05676931) and anti-adenosine agent Etrumadenant (AB928) (NCT04791839). The ARTEMIDE-01 study (NCT04995523) will be further exploring the utility of bi-specific anti-TIGIT/anti-PD-1 antibody AZD2936 in a safety and feasibility study in metastatic NSCLC.

In extensive-stage small cell lung cancer (ES-SCLC), the addition of anti-PD-L1 in two recent randomised trials; CASPIAN [40–42] And Impower 133 has shown survival benefits over chemotherapy alone, albeit modest [36–38]. In the recently reported ASCO data from KEYNOTE-604, which treated ES-SCLC with anti-PD-1 agent, pembrolizumab in combination with Etoposide versus standard of care treatment, again the OS benefit was modest with the combination arm and did not reach significance at final analysis (HR 0.80 [95% CI 0.64–0.98], *P* = 0.0164; median 10.8 vs. 9.7 months) [43]. The immune axis in this disease is thought to be far less PD-1/PD-L1 centric than in NSCLC, and avenues have been forged to seek out other checkpoints to target. SKYSCRAPER-02 (NCT04256421) [44] has recently reported its preliminary results in a phase III randomised setting whereby ES-SCLC patients are treated with Atezolizumab/Carboplatin/Etoposide +/- Tirogolumab (anti-TIGIT). A total of 490 patients were randomised. In the primary and final analysis sets, no additional benefit was seen in OS or PFS with the addition of the anti-TIGIT monoclonal antibody. No additional toxicities were observed in the quad combination arm. The final overall survival analysis is awaited, but the preliminary data from this trial suggest the immune contexture may be radically different in SCLC compared to NSCLC. PD-1 T cell expression is crucial to PD-1/PD-L1 checkpoint blockade and especially in correlation with the DNAM-1-positive activated effector T cell phenotype; low PD-1/PD-L1 expression in SCLC [45–47] is one plausible explanation for the lack of durable response seen in the trials reported above.

## DNAM-1 agonism

DNAM-1 is a critical regulator of the response to dual TIGIT/PD-1 blockade as we have discussed above. Moreover, CD155 cross-linking with DNAM-1 potentiates NK cell cytotoxicity against tumour cells in a range of cancers. It is therefore worth discussing the therapeutic implications of DNAM-1 agonism as a therapeutic strategy in cancer whether it be as a stand-alone strategy or more appropriately in synergy with TIGIT/PD-1 blockade. The Eli-Lilly clinical trial; NCT04099277 tested agonistic anti-DNAM-1 in multiple cancers with and without Pembrolizumab. This study was terminated early but opened a door into exploring this area as a therapeutic avenue. Pre-clinical data has reliably shown the benefits of DNAM-1 agonism [48] in Multiple Sclerosis and Melanoma models. Use of immunomodulatory agent Laquinimod (Quinoline-2-Carboxamide) activated NK cells via the Aryl Hydrocarbon receptor pathway with increase in DNAM-1 surface expression in murine models. This resulted in improved NK-mediated cytotoxicity against B16F10 melanoma cells and augmented immunoregulatory functions in experimental allergic encephalomyelitis by interacting with CD155+ dendritic cells (DC). The immunosuppressive effect of Laquinimod-activated NK cells was due to decreasing MHC class II antigen presentation by DC and not by increasing DC killing [48].

## Future directions

Targeting the PD-1/PD-L1 checkpoint has occupied a stable space in the treatment of advanced NSCLC and this is still changing; the combination of nivolumab and ipilimumab demonstrated overall survival benefit in the first-line setting, regardless of PD-L1 status and Tumour Mutational Burden (Checkmate-227) [49]. However, given the pre-clinical data and early-phase randomised data discussed above, several exciting new compounds [39, 50] targeting TIGIT are currently in clinical trials and in preclinical development. These agents, when combined with PD-1/PD-L1 inhibition, seem to confer higher response rates compared to PD-1/PD-L1 inhibition alone. Questions remain regarding the setting of disease (anti-PD-1/PD-L1 naive or refractory), the combination of treatment regimens and which biomarkers will help stratify disease response (PD-L1, TIGIT, DNAM-1, components of the TME). If this can be confirmed in larger trials, the treatment paradigm and regimen will evolve dramatically and the quest for new targets (LAG3, OX40, IDO), vaccine development and harnessing the TME will certainly be of vital importance in treatment-refractory and resistant patients.

## Data Availability

Data derived from public domain sources.

## Abbreviations

ES-SCLC: Extensive-stage small cell lung cancer
ICD: ITIM-containing intracellular domain
Ig: Immunoglobulin
ITIM: Inhibitory motif
ITT: Ig tail-tyrosine
LAG-3: Lymphocyte-activation gene 3
NK: Natural killer
NSCLC: Non-small cell lung cancer
ORR: Objective response rate
PFS: Progression-free survival
TRAE: Treatment-related adverse events
SCLC: Small cell lung cancer
TIGIT: T cell immunoglobulin and ITIM domain
T_regs_: T_regulatory_
TILs: Tumour-infiltrating lymphocytes
TIM-3: T-cell immunoglobulin and mucin-domain containing-3
TNM: Tumour/Nodal/Metastasis
TME: Tumour microenvironment
anti-TIGIT: Atezolizumab/Carboplatin/Etoposide +/- Tirogolumab
